# Robust Fault Estimation Using the Intermediate Observer: Application to the Quadcopter

**DOI:** 10.3390/s20174917

**Published:** 2020-08-31

**Authors:** Ngoc Phi Nguyen, Tuan Tu Huynh, Xuan Phu Do, Nguyen Xuan Mung, Sung Kyung Hong

**Affiliations:** 1 Department of Aerospace Engineering, Sejong University, Seoul 143-747 (05006), Korea; npnguyen@sejong.ac.kr (N.P.N.); xuanmung1009@gmail.com (N.X.M.); 2 Department of Electrical Engineering, Yuan Ze University, No. 135, Yuandong Road, Zhongli, Taoyuan 320, Taiwan; huynhtuantu@saturn.yzu.edu.tw; 3 Department of Electrical Electronic and Mechanical Engineering, Lac Hong University, No. 10, Huynh Van Nghe Road, Bien Hoa, Dong Nai 810000, Vietnam; 4 MediRobotics Laboratory, Department of Machatronics and Sensor Systems Technology, Vietnamese-German University, Binh Duong 820000, Vietnam; phu.dx@vgu.edu.vn

**Keywords:** fault diagnosis, quadrotor, UAVs, sliding mode observer, sensor fault, quadcopter

## Abstract

In this paper, an actuator fault estimation technique is proposed for quadcopters under uncertainties. In previous studies, matching conditions were required for the observer design, but they were found to be complex for solving linear matrix inequalities (LMIs). To overcome these limitations, in this study, an improved intermediate estimator algorithm was applied to the quadcopter model, which can be used to estimate actuator faults and system states. The system stability was validated using Lyapunov theory. It was shown that system errors are uniformly ultimately bounded. To increase the accuracy of the proposed fault estimation algorithm, a magnitude order balance method was applied. Experiments were verified with four scenarios to show the effectiveness of the proposed algorithm. Two first scenarios were compared to show the effectiveness of the magnitude order balance method. The remaining scenarios were described to test the reliability of the presented method in the presence of multiple actuator faults. Different from previous studies on observer-based fault estimation, this proposal not only can estimate the fault magnitude of the roll, pitch, yaw, and thrust channel, but also can estimate the loss of control effectiveness of each actuator under uncertainties.

## 1. Introduction

Unmanned aerial vehicles (UAVs) have attracted attention over many years owing to their vital achievements and significant advantages in various applications, such as rescue [1,2], coastal surveillance [3,4], forest monitoring [5,6], military, defense [7,8], and robust control with uncertainties [9,10,11]. In comparison with manned aerial vehicles, the control of UAVs is more complex since all tasks are operated autonomously through an embedded flight controller or by a pilot.

Quadcopter, a type of UAV system, has been used and developed for various applications owing to its numerous advantages, such as simplicity, small size, indoor and outdoor operation, and agility, which have rendered it more famous than other types of UAV systems. Quadcopters have been widely investigated and developed for different tasks and environments, including tracking control, formation flight, object tracking, remote sensing, and fault-tolerant control (FTC).

In recent years, studies on FTC have received significant study in the scientific community, which could further increase the reliability of the quadcopter during flights. Normally, an FTC can be classified as a passive FTC (PFTC) or an active FTC (AFTC). PFTC techniques have been extensively used in previous studies [12,13,14], which have the advantage that fault identification is not required in controller design. However, these approaches have a lower fault-tolerance capability. To deal with this issue, AFTC approaches have been proposed to provide improved fault-tolerance capability and condition-based control for flight systems. In these schemes, fault diagnosis (FD) is an essential precondition to identify the magnitude and location of faults, which is combined with a nominal controller to accommodate the effect of faults. Moreover, through FD information, the control law allows for a landing action in an emergency case. As a result, FD is a major feature in the design of the AFTC system.

The problem with the FD approach has been addressed in several studies. In [15,16,17], a model- based FD was investigated to monitor the sensor fault and actuator fault, which was validated through a set of observer residuals; however, this algorithm is inaccurate and unsuitable for the isolation and identification of faults. In the same context, a polynomial observer was developed to estimate actuator faults. In [18], Aguilar-Sierra et al. proposed an FD technique to estimate actuator faults using a polynomial observer. In [19], an FD method based on a Kalman filter technique was investigated for actuator faults presenting experimental results; but this method lacks robustness against disturbance. In [20], a linear parameter varying technique was developed for a quadrotor helicopter to estimate the faults. Using this fault estimation information, a fault-tolerant controller was designed to accommodate faults. In [21,22], Cen et al. proposed an adaptive law for Thau observer to estimate the time-varying actuator faults in a quadrotor. The FD strategies presented in the above studies show either valuable simulation or experimental results. However, these methodologies do not examine the uncertainties in their mathematical models.

To address the above statement, several effective strategies, such as high-gain observer [23], neural network [24,25], and nonlinear descriptor observer [26], were previously proposed for nonlinear systems, but none of these methods concentrated on the quadcopter model or real experimental tests. Only a few studies investigated the fault diagnosis problem in quadcopter platforms. In [27], actuator fault diagnosis approaches based on fuzzy techniques were investigated in the quadcopter model showing simulation results. In [28], the adaptive observer based on $H_{\infty }$ was proposed for fault estimation considering the simulation and experimental results. Other effective methods, such as sliding mode observers (SMOs) have been developed to enhance the robustness of fault identification and time convergence. In this sense, SMO based on a linear parametric varying technique was proposed for actuator fault reconstruction [29]. In [30], a sliding- mode observer combined with Thau observer was proposed to estimate the magnitude of the fault showing experimental works. However, the current studies on quadcopters are still based on assumptions or observer-matching conditions.

Motivated by the above challenges, this article proposes a fault estimation strategy for a quadcopter system with relaxing matching conditions. To be more specific, an intermediate variable combined with an intermediate observer has been improved to estimate both states and faults. The stability of the proposed algorithm was proved using the Lyapunov theory. It was shown that the error system is uniformly ultimately bounded. In contrast to previous results, the main contributions of this article are as follows:

- An intermediate estimator is provided in the quadcopter system to estimate states and faults in the presence of uncertainties. The proposed method aims to relax the matching conditions or equation constraints.
- Stability analysis is described to validate the convergence of the error system.
- The experimental works on the quadcopter are demonstrated to validate the proposed algorithm.

The remainder of this article is organized as follows. Section 2 provides the mathematical model of the quadcopter system. In Section 3, the proposed FD method is introduced. Experimental results are described in Section 4. The conclusions and further works are stated in Section 5.

## 2. Mathematical Model of the Quadcopter

**Figure 1:**
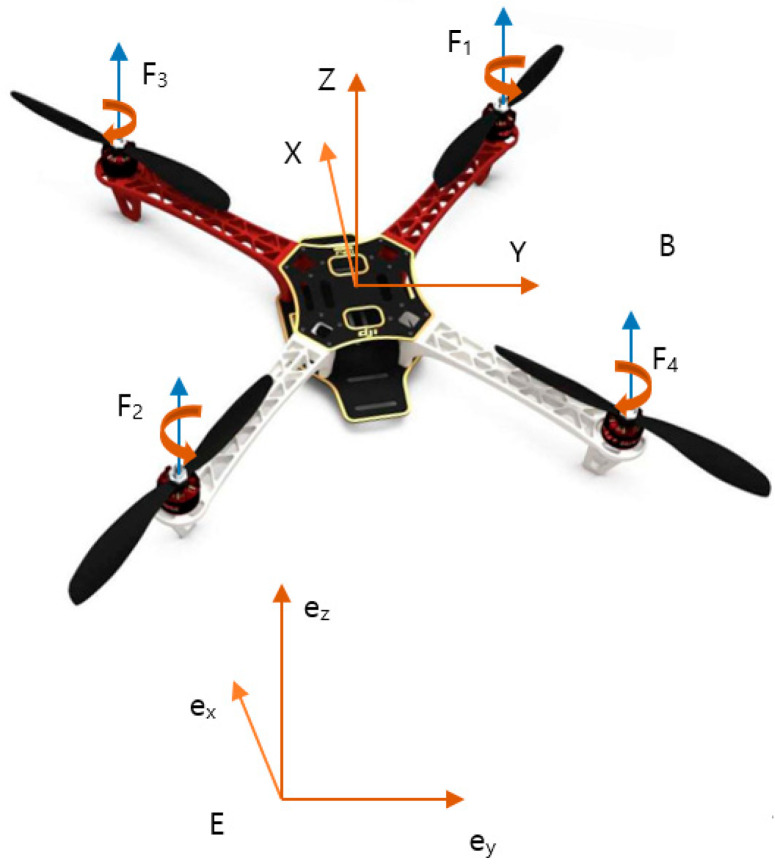
Quadcopter model.

The body frame B and inertial frame E are used to present the dynamics of the quadcopter, as shown in Figure 1. In the body frame, the XY plane is located at the surface, and the *Z*-axis follows the right-hand rule. In the quadcopter system, the center of gravity is located at the origin of the body frame. The rotation matrix is defined to transform from the body frame to the inertial frame as

(1) $$R(\phi ,\theta ,\psi )=\left[\begin{array}{ccc} c\psi c\theta & c\psi s\theta s\phi -s\psi c\phi & c\psi s\theta c\phi +s\psi c\phi \\ s\psi c\theta & s\psi s\theta s\phi +c\psi c\phi & s\psi s\theta c\phi -s\phi c\psi \\ -s\theta & c\theta s\psi & c\theta c\theta \end{array}\right]$$

where s and c denote sin and cos; ϕ,θ, ψ denote Euler angles.

The quadcopter includes two motors (1, 2) rotating in a counterclockwise direction and the remaining two motors rotating clockwise. The four control variables are expressed as:

(2) $$\left\{ \begin{array}{l} U_{1}=F_{1}+F_{2}+F_{3}+F_{4}\\ U_{2}=\left(-F_{1}+F_{2}+F_{3}-F_{4}\right)L\sqrt{2}/2\\ U_{3}=(F_{1}-F_{2}+F_{3}-F_{4})L\sqrt{2}/2\\ U_{4}=\tau_{1}+\tau_{2}-\tau_{3}-\tau_{4} \end{array}\right.$$

where $\tau_{i}\approx K_{d}u_{i}$ and $F_{i}\approx K_{th}u_{i}$ denote the respective torques and forces produced by the ith motor; $u_{i}$ is the pulse-width modulation; L is the arm length; $U_{1}$ is the thrust force; and $U_{2},\;U_{3},\;U_{4}$ are the torques in the directions ϕ,  θ,  ψ, respectively.

Equation (2) can be rewritten as

(3) $$\left[\begin{array}{l} U_{2}\\ U_{3}\\ U_{4}\\ U_{1} \end{array}\right]=\left[\begin{array}{cccc} -K_{th}L\sqrt{2}/2 & K_{th}L\sqrt{2}/2 & K_{th}L\sqrt{2}/2 & -K_{th}L\sqrt{2}/2\\ K_{th}L\sqrt{2}/2 & -K_{th}L\sqrt{2}/2 & K_{th}L\sqrt{2}/2 & -K_{th}L\sqrt{2}/2\\ K_{d} & K_{d} & -K_{d} & -K_{d}\\ K_{th} & K_{th} & K_{th} & K_{th} \end{array}\right]\left[\begin{array}{l} u_{1}\\ u_{2}\\ u_{3}\\ u_{4} \end{array}\right]$$

The body dynamics are described using the Newton–Euler equation as [31]:

(4) $$\left\{ \begin{array}{l} m\ddot{r}=R\left[\begin{array}{l} 0\\ 0\\ U_{1} \end{array}\right]-\left[\begin{array}{l} 0\\ 0\\ mg \end{array}\right]-\dot{\omega }\times m\ddot{r}\\ I\ddot{\omega }=\left[\begin{array}{l} U_{2}\\ U_{3}\\ U_{4} \end{array}\right]-\dot{\omega }\times I\ddot{\omega } \end{array}\right.$$

where m is the total mass; g is the gravity; ω and I are the angular velocity and the inertia vector, respectively; r is the position in an inertial frame.

The dynamics of the quadcopter can be presented through six equations [32]:

(5) $$\left\{ \begin{array}{l} \ddot{x}=\left\{U_{1}(\cos\phi \sin\theta \cos\psi +\sin\phi \sin\psi )-K_{x}\dot{x}\right\}/m\\ \ddot{y}=\left\{U_{1}(\cos\phi \sin\theta \sin\psi -\sin\phi \cos\psi )-K_{y}\dot{y}\right\}/m\\ \ddot{z}=-g+\left\{U_{1}(\cos\phi \cos\theta )-K_{z}\dot{z}\right\}/m\\ \ddot{\phi }=\left(U_{2}+(I_{2}-I_{3})\dot{\theta }\dot{\psi }-K_{\phi }\dot{\phi }\right)/I_{1}\\ \ddot{\theta }=\left(U_{3}+(I_{3}-I_{1})\dot{\phi }\dot{\psi }-K_{\theta }\dot{\theta }\right)/I_{2}\\ \ddot{\psi }=\left(U_{4}+(I_{1}-I_{2})\dot{\phi }\dot{\theta }-K_{\psi }\dot{\psi }\right)/I_{3} \end{array}\right.$$

where $I_{1},\;\;I_{2},\;\;I_{3}$ represent the moment inertia along the x,  y,  z axes; and $K_{\phi },\;\;K_{\theta },\;\;K_{\psi },\;K_{x},\;K_{y}$ represent drag coefficients.

Let $x^{T}=\left[\begin{array}{cccccc} \phi & \theta & \psi & \dot{\phi } & \dot{\theta } & \dot{\psi } \end{array}\right]$ be the state vector, $u^{T}=\left[\begin{array}{cccc} U_{\phi } & U_{\theta } & U_{\psi } & U_{T} \end{array}\right]$ be the control input vector, and $y=\left[\begin{array}{cccccc} \phi & \theta & \psi & \dot{\phi } & \dot{\theta } & \dot{\psi } \end{array}\right]$ be the output vector. Equation (4) is rewritten as

(6) $$\left\{ \begin{array}{l} \dot{x}(t)=Ax(t)+Bu(t)+N\xi (t,x(t))+G\\ y(t)=Cx(t) \end{array}\right.$$

where $N=I_{4\times 4}$ is the distribution matrix d(t) denotes the disturbance vector; $A=\left[\begin{array}{cc} 0_{4\times 4} & I_{4\times 4}\\ 0_{4\times 4} & 0_{4\times 4} \end{array}\right]$, $C=I_{8\times 8}$, $B={\left[\begin{array}{cccccccc} 0 & 0 & 0 & 0 & 1 & 0 & 0 & 0\\ 0 & 0 & 0 & 0 & 0 & 1 & 0 & 0\\ 0 & 0 & 0 & 0 & 0 & 0 & 1 & 0\\ 0 & 0 & 0 & 0 & 0 & 0 & 0 & 1 \end{array}\right]}^{T}$, $\xi (t,x(t))=\left[\begin{array}{l} (\dot{\theta }\dot{\psi }(I_{2}-I_{3})-{\bar{J}}_{m}\dot{\theta }\Omega )/I_{1}\\ (\dot{\phi }\dot{\psi }(I_{3}-I_{1})-{\bar{J}}_{m}\dot{\phi }\Omega )/I_{2}\\ \dot{\phi }\dot{\theta }(I_{1}-I_{2})/I_{3}\\ 0 \end{array}\right]$; $G={\left[\begin{array}{cccccccc} 0 & 0 & 0 & 0 & 0 & 0 & 0 & -g \end{array}\right]}^{T}$; $U_{\phi }=U_{2}/I_{1};\;\;U_{\theta }=U_{3}/I_{2};\;\;U_{\psi }=U_{4}/I_{3};\;\;U_{T}=U_{1}/m$.

## 3. Methodology

The aim of this section is to provide the fault estimation technique based on the intermediate observer and reducing fault estimation error method for the quadcopter system. In detail, a robust intermediate observer is presented in Section 3.1 to estimate the fault magnitude of the roll, pitch, yaw, and thrust motion. In Section 3.2, a magnitude order balance method is applied to reduce the fault estimation error, which is caused by an imbalance magnitude problem of the yaw motion. Moreover, this section also presents a technique to obtain the loss of control effectiveness (LoCE) of each actuator through the presented method in Section 3.1.

### 3.1. Design of the Intermediate Observer

When a fault occurs, Equation (6) can be written as:

(7) $$\left\{ \begin{array}{l} \dot{x}(t)=Ax(t)+Bu(t)+N\xi (t,x(t))+G+E_{a}f_{a}(t)\\ y(t)=Cx(t) \end{array}\right.$$

where $E_{a}$ is the fault matrix, $f_{a}(t)$ is the fault vector.

To design a robust intermediate observer [33], some assumptions need to be considered.

**Assumption** **1.** *The unknown disturbance is norm-bounded with constant*$\theta_{1}$*, i.e.,*$\left\|\xi (t,x(t))\right\|\leq \theta_{1}$*, where*$0\leq \theta_{1}\leq \infty$.

**Assumption** **2.** *The first derivative of*$f_{a}(t)$*satisfies*$\left\|{\dot{f}}_{a}(t)\right\|\leq \theta_{2}$*, with*$0\leq \theta_{2}\leq \infty$.

**Assumption** **3.** $E_{a}$ *has a full column rank.*

**Assumption** **4.** *There exists a complex number* λ *with non-negative real part that satisfies*

(8) $$rank\left[\begin{array}{cc} A-\lambda I & E_{a}\\ C & 0 \end{array}\right]=n+rank(E_{a})$$

An auxiliary variable is constructed as

(9) $$\tau =f_{a}(t)-Sx(t)$$

where $S=\alpha E_{a}{}^{T}$; α is a constant that needs to be chosen.

From Equations (7) and (9), we obtain

(10) $$\dot{\tau }={\dot{f}}_{a}-S\left(Ax(t)+Bu+E_{a}\tau +E_{a}Sx(t)+N\xi (t,x(t)+G)\right)$$

The intermediate estimator is introduced as:

(11) $$\dot{\hat{x}}(t)=A\hat{x}(t)+Bu(t)+E_{a}{\hat{f}}_{a}(t)+G+L(y(t)-\hat{y}(t))$$

(12) $$\dot{\hat{\tau }}(t)=-S\left(A\hat{x}(t)+Bu(t)+E_{a}\hat{\tau }(t)+E_{a}S\hat{x}(t)+G\right)$$

(13) $$\hat{y}(t)=C\hat{x}(t)$$

(14) $${\hat{f}}_{a}(t)=\hat{\tau }(t)+S\hat{x}(t)$$

where $\hat{x}(t),\;\;\hat{\tau }(t),\;\;\hat{y}(t),{\hat{f}}_{a}(t)$ are the state observer vector, intermediate estimator, output observer vector, and fault estimation of $f_{a}(t)$, respectively.

Let $e_{x}=x(t)-\hat{x}(t)$ and $e_{f}(t)=f_{a}(t)-{\hat{f}}_{a}(t)$. The error system is described as

(15) $${\dot{e}}_{x}=(A-LC)e_{x}+E_{a}e_{f}+N\xi (t,x(t))$$

(16) $${\dot{e}}_{\tau }={\dot{f}}_{a}-\alpha E^{T}(Ae_{x}+E_{a}e_{\tau }+E_{a}Se_{x}+N\xi (t,x(t)))$$

**Theorem** **1.** *If we apply the intermediate estimator Equations (11)–(14) to state-space model (7) and using Assumptions 1–4, and there exist scalars*α>0,  ε>0*and matrix*L*such that*

(17) $$\left[\begin{array}{cc} \Pi_{11} & \Pi_{12}\\ {}* & \Pi_{22} \end{array}\right]<0$$

*where*$\Pi_{11}={\left(A-LC\right)}^{T}+\left(A-LC\right)+2\alpha E_{a}E_{a}^{T}+\frac{1}{\varepsilon }NN^{T}$*,*$\Pi_{12}=E-\mu \alpha A^{T}E_{a}-\mu \alpha^{2}E_{a}E_{a}^{T}E_{a}$, *then*$\Pi_{22}=\frac{\mu }{\varepsilon }-2\mu \alpha E_{a}^{T}E_{a}+\frac{\mu \alpha }{\varepsilon }E_{a}^{T}NN^{T}E_{a}$.

Then, the error systems (15) and (16) are uniformly ultimately bounded.

**Proof.**  Choosing the Lyapunov function as:

(18) $$V=e_{x}^{T}e_{x}+\mu e_{\tau }^{T}e_{\tau }$$

The first derivative of V(t) is given as:

(19) $$\begin{array}{ll} \dot{V} & ={\dot{e}}_{x}^{T}e_{x}+e_{x}^{T}{\dot{e}}_{x}+{\dot{e}}_{\tau }^{T}e_{\tau }+e_{\tau }^{T}{\dot{e}}_{\tau }\\ & ={\left[\left(A-LC)e_{x}+E_{a}e_{f}+N\xi (t,x(t)\right)\right]}^{T}e_{x}\\ & +e_{x}^{T}\left[\left(A-LC)e_{x}+E_{a}e_{f}+N\xi (t,x(t)\right)\right]\\ & +\mu {\left[{\dot{f}}_{a}-S(Ae_{x}+E_{a}e_{\tau }+E_{a}Se_{x}+N\xi (t,x(t))\right]}^{T}e_{\tau }\\ & +\mu e_{\tau }^{T}\left[{\dot{f}}_{a}-S(Ae_{x}+E_{a}e_{\tau }+E_{a}Se_{x}+N\xi (t,x(t))\right]\\ & =e_{x}^{T}\left[{\left(A-LC\right)}^{T}+(A-LC)\right]e_{x}+2e_{x}^{T}E_{a}e_{f}\\ & +2e_{x}^{T}N\xi (t,x(t))\;+2\mu e_{\tau }^{T}{\dot{f}}_{a}-2\mu \alpha e_{\tau }^{T}E_{a}^{T}(A+\alpha E_{a}E_{a}^{T})e_{x}\\ & -2\mu \alpha e_{\tau }^{T}E_{a}^{T}E_{a}e_{\tau }\;-2\mu \alpha e_{\tau }^{T}E^{T}N\xi (t,x(t)) \end{array}$$

Using the Lemma, we obtain

(20) $$2e_{x}^{T}N\xi (t,x(t))\leq \frac{1}{\varepsilon }e_{x}^{T}NN^{T}e_{x}+\varepsilon \theta_{1}^{2}$$

(21) $$2e_{\tau }^{T}\dot{f}\leq \frac{1}{\varepsilon }e_{\tau }^{T}e_{\tau }+\varepsilon {\bar{\theta }}_{2}^{2}$$

(22) $$-2e_{\tau }^{T}E_{a}^{T}N\xi (t,x(t))\leq \frac{1}{\varepsilon }e_{\tau }^{T}E_{a}^{T}NN^{T}E_{a}+\varepsilon {\bar{\theta }}_{1}^{2}$$

From (19)–(22) and $e_{f}=e_{\tau }+Se_{x}=e_{\tau }+\alpha E^{T}e_{x}$, we obtain

(23) $$\begin{array}{ll} \dot{V} & =e_{x}^{T}\left[{\left(A-LC\right)}^{T}+(A-LC)\right]e_{x}+2e_{x}^{T}E_{a}e_{\tau }+2\alpha e_{x}^{T}E_{a}E_{a}^{T}e_{x}+\frac{1}{\varepsilon }e_{x}^{T}NN^{T}e_{x}\\ & +\frac{\mu }{\varepsilon }e_{\tau }^{T}e_{\tau }-2\mu \alpha e_{\tau }^{T}E_{a}^{T}(A+\alpha E_{a}E_{a}^{T})e_{x}-2\mu \alpha e_{\tau }^{T}E_{a}^{T}E_{a}e_{\tau }\\ & +\frac{\mu \alpha }{\varepsilon }e_{\tau }^{T}E_{a}^{T}NN^{T}E_{a}e_{\tau }+\varepsilon (1+\mu \alpha ){\bar{\theta }}_{1}^{2}+\mu \varepsilon {\bar{\theta }}_{2}^{2} \end{array}$$

Let us define $\tilde{e}(t)={\left[\begin{array}{cc} e_{x}^{T} & e_{\tau }^{T} \end{array}\right]}^{T}$, then, Equation (23) becomes

(24) $$\dot{V}\leq {\tilde{e}}^{T}\Sigma_{1}{\tilde{e}}^{T}+\varepsilon (1+\mu \alpha ){\bar{\theta }}_{1}^{2}+\mu \varepsilon {\bar{\theta }}_{2}^{2}$$

where

(25) $$\Sigma_{1}=\left[\begin{array}{cc} \Sigma_{11} & \Sigma_{12}\\ {}* & \Sigma_{22} \end{array}\right]$$

with

(26) $$\Sigma_{11}={\left(A-LC\right)}^{T}+\left(A-LC\right)+2\alpha E_{a}E_{a}^{T}+\frac{1}{\varepsilon }NN^{T}$$

(27) $$\Sigma_{12}=E_{a}-\mu \alpha A^{T}E_{a}-\mu \alpha^{2}E_{a}E_{a}^{T}E_{a}$$

(28) $$\Sigma_{22}=\frac{\mu }{\varepsilon }I_{n}-2\mu \alpha E_{a}^{T}E_{a}+\frac{\mu \alpha }{\varepsilon }E_{a}^{T}NN^{T}E_{a}$$

If the following inequality holds,

(29) $$\left[\begin{array}{cc} \Sigma_{11} & \Sigma_{12}\\ {}* & \Sigma_{22} \end{array}\right]<0$$

then, one can achieve $\dot{V}\left(t\right)<-\sigma {\left\|\tilde{e}(t)\right\|}^{2}+\eta$, where $\eta =\varepsilon (1+\mu \alpha ){\bar{\theta }}_{1}^{2}+\mu \varepsilon {\bar{\theta }}_{2}^{2}$ and $\sigma =\lambda_{\min}(-\Sigma_{1})$. It follows that $\dot{V}\left(t\right)<0$ for $\sigma {\left\|\tilde{e}(t)\right\|}^{2}>\eta$, which indicates that $\left(e_{x}(t),e_{\tau }(t)\right)$ converges to a small set according to the Lyapunov theory. Therefore, the system error is uniformly ultimately bounded. □

### 3.2. Magnitude Order Balance and Fault Estimation of Each Actuator

The control input vector $u={\left[\begin{array}{cccc} U_{\phi } & U_{\theta } & U_{\psi } & U_{T} \end{array}\right]}^{T}$ is obtained from thrusts and torques in Equation (6), and is used to estimate the actuator fault $f_{a}={\left[\begin{array}{cccc} f_{\phi } & f_{\theta } & f_{\psi } & f_{T} \end{array}\right]}^{T}$ using the intermediate observer. However, the fault estimation from the yaw motion has a large error compared with the roll, pitch, and thrust motions due to the imbalance of the magnitude order [21]. To overcome this issue, the magnitude order of the four channels should be adjusted in the same range by using the following modified control input vector

(30) $$u={\left[\begin{array}{cccc} U_{\phi } & U_{\theta } & \upsilon U_{\psi } & U_{T} \end{array}\right]}^{T}$$

where υ are adjustment gains.

**Theorem** **2.** *Assume that the new estimation values* $\;\;{\hat{f}}_{\psi }^{*}$ *are obtained from the intermediate observer and the estimation values of* $f_{\psi }$ *do not change. If the former fault estimation of yaw motion is denoted by* $\;{\hat{f}}_{\psi }$ *, then the following relationship should hold:*

(31) $${\hat{f}}_{\psi }^{*}=\upsilon {\hat{f}}_{\psi }$$

**Proof.**  Recall the intermediate observer design

(32) $$\dot{\hat{x}}(t)=A\hat{x}(t)+Bu(t)+E_{a}{\hat{f}}_{a}(t)+L(y(t)-\hat{y}(t))$$

If the error system converges to a small set as mentioned in Section 3.1, and the following requirement is satisfied: $e_{y}=0,$ $\dot{\hat{x}}(t)=0$ and $\hat{x}(t)=0$. The fault estimation vector ${\hat{f}}_{a}(t)$ can be obtained from

(33) $$Bu(t)+E_{a}{\hat{f}}_{a}(t)=0$$

For the yaw motion, Equation (12) is described as

(34) $$U_{\psi }+{\hat{f}}_{\psi }(t)=0$$

If $U_{\psi }^{*}=\upsilon U_{\psi }$ then ${\hat{f}}_{\psi }^{*}=\upsilon {\hat{f}}_{\psi }$.

When the new fault estimation algorithm is achieved, the desired real fault estimation can be achieved as

(35) $${\hat{f}}_{\psi }={\hat{f}}_{\psi }^{*}/\upsilon$$

□

**Remark** **1.** *The modification of the control input vector using the adjustment gain factor technique does not affect the intermediate observer. However, it is applied to handle the magnitude order unbalance issue and reduce the fault estimation errors, which is discussed in more detail in the experimental section described in Section 4.*

To estimate the fault magnitude of each motor, the dynamic system (7) is rewritten as

(36) $$\left\{ \begin{array}{l} \dot{x}(t)=Ax(t)+B_{1}u^{*}(t)-B_{1}\Gamma \Lambda +N\xi (t,x(t))+G\\ y(t)=Cx(t) \end{array}\right.$$

where $B_{1}={\left[\begin{array}{cccccccc} 0 & 0 & 0 & 0 & -\frac{K_{th}L\sqrt{2}}{2I_{1}} & \frac{K_{th}L\sqrt{2}}{2I_{2}} & \frac{K_{d}}{I_{3}} & K_{th}\\ 0 & 0 & 0 & 0 & \frac{K_{th}L\sqrt{2}}{2I_{1}} & -\frac{K_{th}L\sqrt{2}}{2I_{2}} & \frac{K_{d}}{I_{3}} & K_{th}\\ 0 & 0 & 0 & 0 & \frac{K_{th}L\sqrt{2}}{2I_{1}} & \frac{K_{th}L\sqrt{2}}{2I_{2}} & -\frac{K_{d}}{I_{3}} & K_{th}\\ 0 & 0 & 0 & 0 & -\frac{K_{th}L\sqrt{2}}{2I_{1}} & -\frac{K_{th}L\sqrt{2}}{2I_{2}} & -\frac{K_{d}}{I_{3}} & K_{th} \end{array}\right]}^{T}$; $\Gamma =\left[\begin{array}{cccc} u_{1} & 0 & 0 & 0\\ 0 & u_{2} & 0 & 0\\ 0 & 0 & u_{3} & 0\\ 0 & 0 & 0 & u_{4} \end{array}\right]$; $u^{*}=\left[\begin{array}{c} u_{1}\\ u_{2}\\ u_{3}\\ u_{4} \end{array}\right]$; $\Lambda ={\left[\begin{array}{cccc} \gamma_{1} & \gamma_{2} & \gamma_{3} & \gamma_{4} \end{array}\right]}^{T}$ is the control effectiveness matrix with $0\leq \gamma_{i}\leq 1;\;\;i=1,2,3,4$; $\gamma_{i}=0\;$ and $\gamma_{i}=1$ indicate that the ith actuator is fault-free and fully damaged, respectively.

Because $Bu(t)=B_{1}u^{*}(t)$ and Equation (36) can be written as:

(37) $$\left\{ \begin{array}{l} \dot{x}(t)=Ax(t)+Bu(t)-B\Pi \Gamma \Lambda +N\xi (t,x(t))+G\\ y(t)=Cx(t) \end{array}\right.$$

where $\Pi =\left[\begin{array}{cccc} -K_{th}L\sqrt{2}/2 & K_{th}L\sqrt{2}/2 & K_{th}L\sqrt{2}/2 & -K_{th}L\sqrt{2}/2\\ K_{th}L\sqrt{2}/2 & -K_{th}L\sqrt{2}/2 & K_{th}L\sqrt{2}/2 & -K_{th}L\sqrt{2}/2\\ K_{d} & K_{d} & -K_{d} & -K_{d}\\ K_{th} & K_{th} & K_{th} & K_{th} \end{array}\right]$.

From Equations (7) and (37), we can obtain $E_{a}f_{a}=-B\Pi \Gamma \Lambda$. In the quadcopter model, we can design $E_{a}=B$. Therefore, LoCE of each actuator (fault estimation of each actuator) can be obtained as

(38) $$\Lambda =-\Gamma^{-1}\Pi^{-1}f_{a}$$

It should be noted that this method not only estimates the fault magnitude of the roll, pitch, yaw, and thrust motion through Equation (14) but also presents the LoCE of each actuator through Equation (38). The results of these equations are discussed in Section 4.

## 4. Experimental Results and Discussion

### 4.1. Experiment Setup

The DJI450 quadcopter frame is used for flight tests in an outdoor environment. The intermediate observer algorithm in the previous section was implemented and tested on a Pixhawk2 using a C++ program executed on the Eclipse environment [34]. The firmware version 3.5.7 is used as an open-source software. The implementation strategy of the proposed fault estimation is presented in Table 1. In the first step, the control inputs from the rotation speeds of the actuator and system matrices in (5) should be obtained. Next, the positive parameter μ is chosen with small values at the beginning to reduce the overestimation problem. Then, the linear matrix inequality (LMI) matrix toolbox from Matlab software is used to find the observer matrix L and constant value of α. After that, the fault estimation of roll, pitch, yaw, and thrust motion in (14) is implemented in the Pixhawk2 flight controller using the result from step 2. If there are some magnitude order imbalance problems, the adjustment gain υ should be chosen in step 4. Finally, LoCE of each actuator in (38) is obtained through (14).

**Figure 2:**
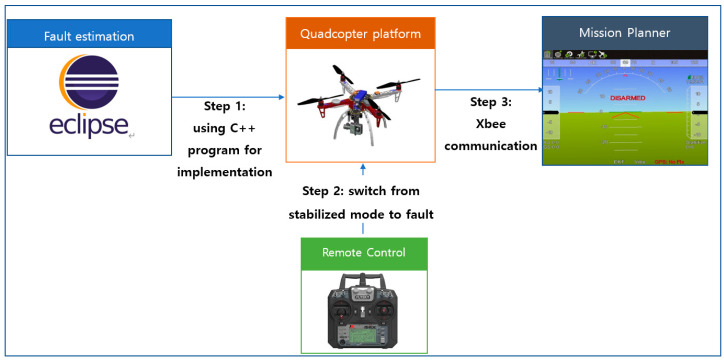
Flight test procedure.

The experimental procedure of the flight test is summarized in Figure 2. First, the fault estimation based on the intermediate observer is implemented on the Pixhawk2 flight controller. Then, to demonstrate the fault scenario, a remote control is used to switch between position hold mode and fault modes, which allows us to apply faults by reducing the pulse width modulation (PWM) of actuators. The Mission Planner software was used to interface the ground station and quadcopter through Xbee telemetry communication [35], which can monitor all system states. The DJI F450 quadcopter parameter and the numerical values of the observer design are presented in Table 2.

The fault in each motor is modeled as the following function:

(39) $$F_{i}=(1-\gamma_{i})K_{th}u_{i}$$

where $\gamma_{i}=0$ indicates the fault-free condition and $\gamma_{i}=1$ indicates the complete motor failure of the ith motor.

### 4.2. Experimental Results

#### 4.2.1. LoCE in One Actuator without Using Magnitude Order Unbalance

**Figure 3:**
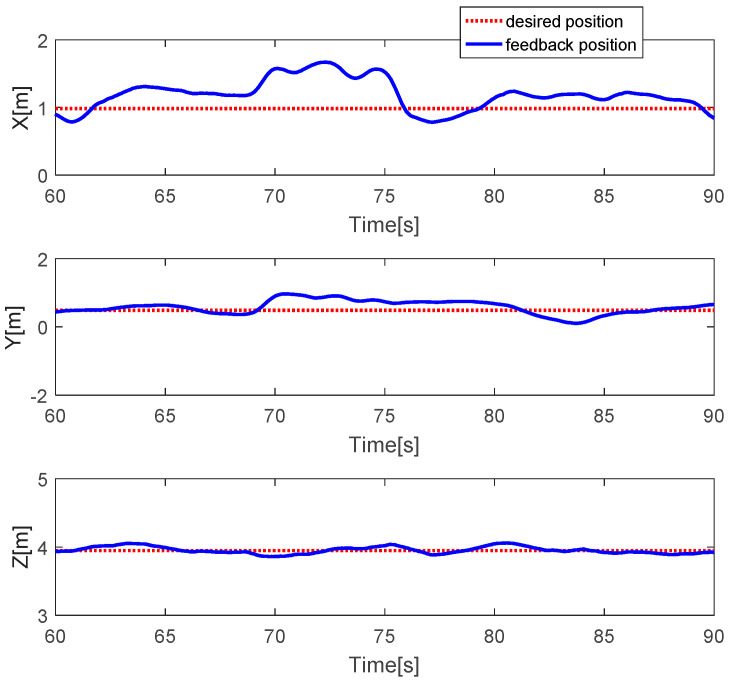
Horizontal and vertical movements of the quadcopter.

**Figure 4:**
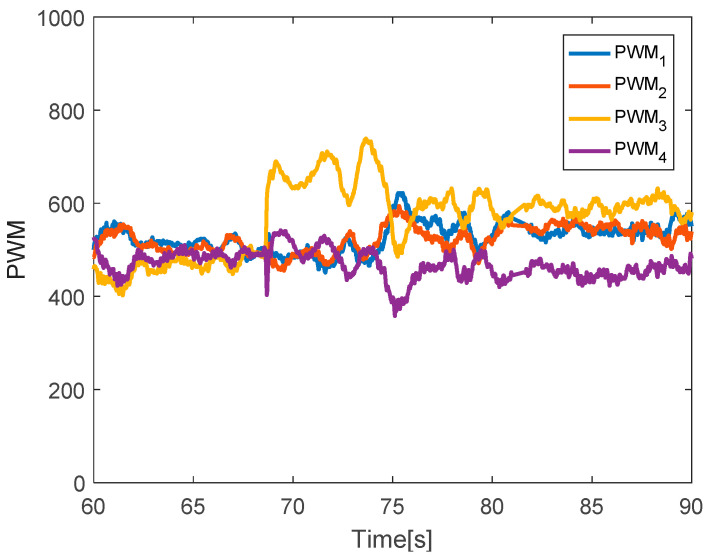
Pulse width modulation (PWM) inputs.

The quadcopter system hovered at the height of 4 m. Faults with different magnitudes were injected into three actuators at time *t* = 68.5 s by 20% LoCE in the third motor. Figure 3 shows that the faults affect the horizontal movement. In detail, the x-direction deviates from the desired position by 60 cm between time *t* = 68.5 s and *t* = 76 s, whereas the y-direction deviates from the desired point by 30 cm. Subsequently, the system is recovered to the desired point due to the PID controller. In the vertical movement, the z-direction has a small deviation from the desired position because the fault magnitude injected in the third direction is small. Figure 4 describes the PWM inputs of all motors. It is shown that, before the fault is injected at *t* = 68.5 s, all PWM inputs have similar values. After faults occur in the third motor, the PID controller can recover the system by increasing the PWM input of the third actuator.

**Figure 5:**
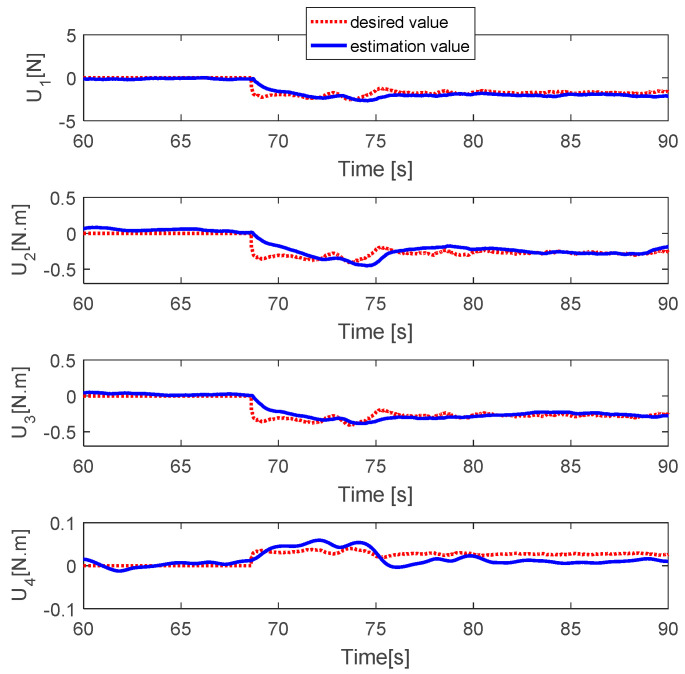
Fault estimations of control inputs.

**Figure 6:**
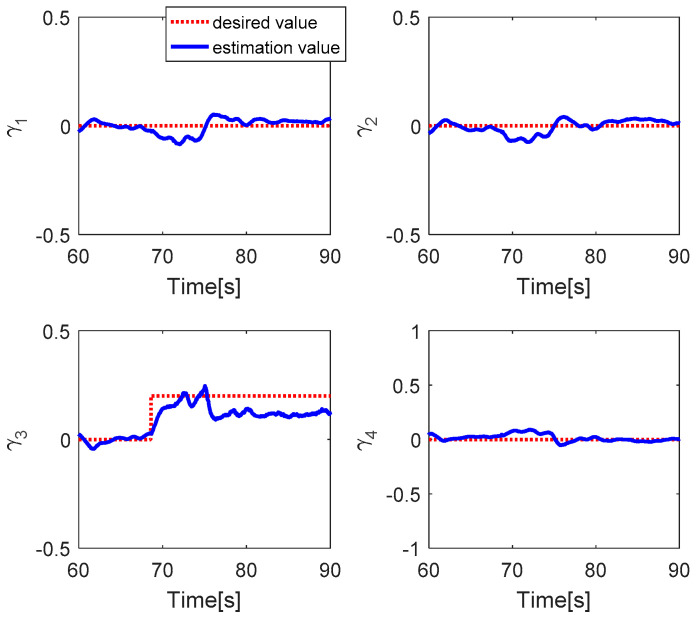
Fault estimation of each actuator.

The fault estimations of the control inputs are presented in Figure 5. This shows that the fault estimation values converge to the desired values in the roll, pitch, and thrust control inputs, but the fault estimation value in the yaw control has a larger error due to the magnitude unbalance problem. Figure 6 shows the fault estimation of each actuator. The fault estimation values of the first, second, and fourth actuators converge to zero. The fault estimation value of the third actuator cannot converge to 0.2 owing to the magnitude unbalance issue.

**Remark** **2.** *It is clear that the fault estimation in the yaw motion has a larger error compared with the other motion due to the magnitude order imbalance problem. To deal with this issue, the magnitude order balance method in Section 3.2 is applied to get a better performance, which is discussed in the next Section 4.2.2, Section 4.2.3 and Section 4.2.4.*

#### 4.2.2. LoCE in One Actuator Using the Magnitude Order Balance Method

**Figure 7:**
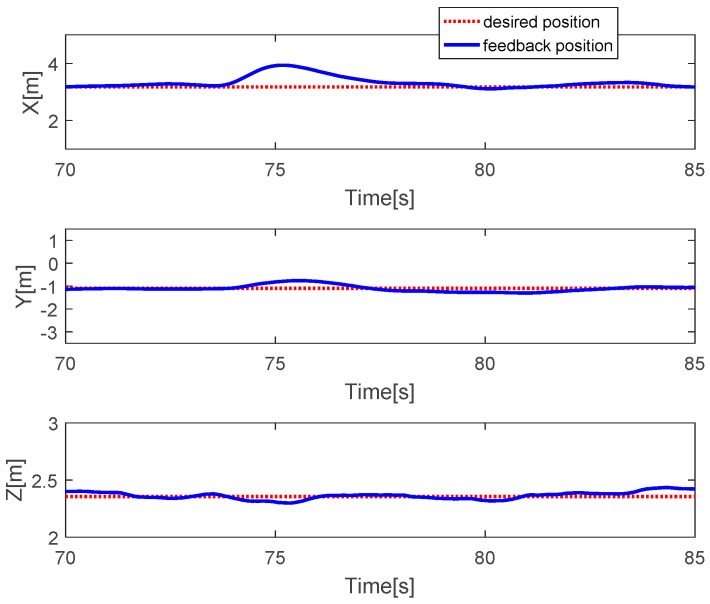
Horizontal and vertical movement of the quadcopter.

**Figure 8:**
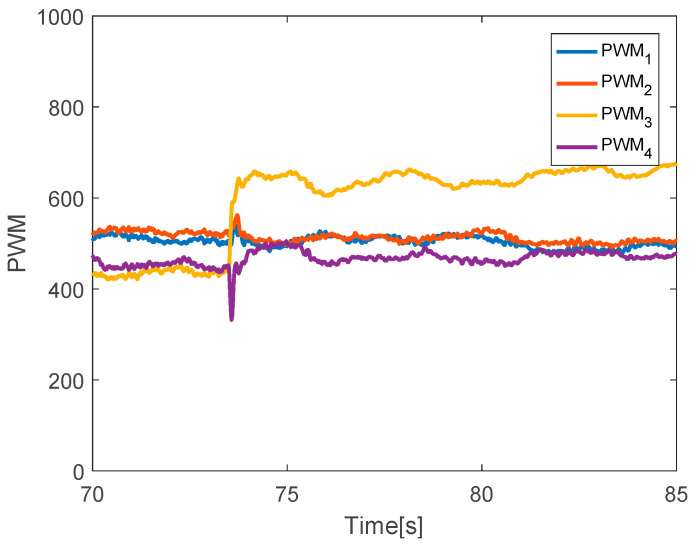
PWM inputs.

The quadcopter system hovers at a height of 2.4 m. The faults with different magnitudes are injected into three actuators at time *t* = 73.5 s by 20% LoCE in the third motor. In the Figure 7, the faults affect horizontal movement. In detail, the x-direction deviates from the desired position by 60 cm between *t* = 73.5 s and *t* = 77.5 s and the y-direction deviates from the desired point by 30 cm. After that, the system is recovered to the desired point due to the PID controller. In the vertical movement, the *z*-direction has a small deviation from the desired position because the fault magnitude injected in the third actuator is small. Figure 8 describes the PWM inputs of all motors. It was shown that before a fault is injected at *t* = 73.5 s, all PWM inputs have similar values. After the faults occur in the third motor, the PID controller can recover the system by increasing the PWM input of the third actuator.

**Figure 9:**
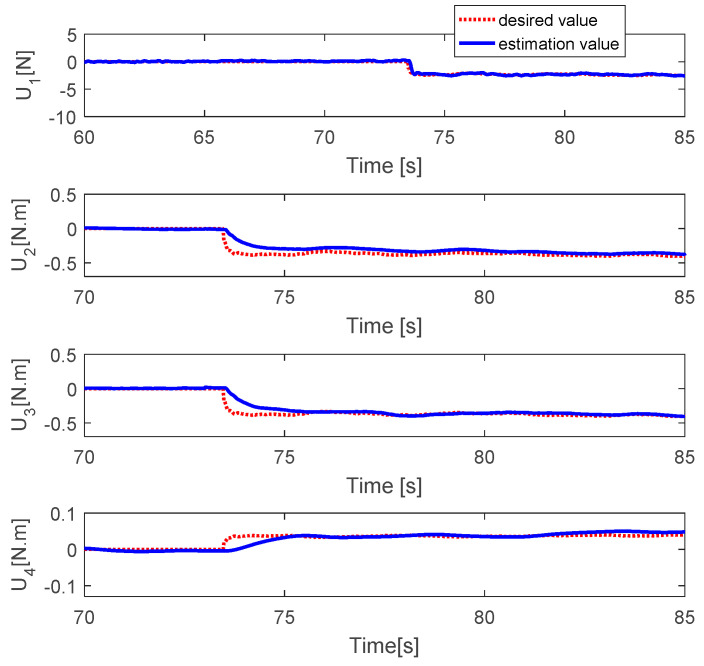
Fault estimation of control inputs.

**Figure 10:**
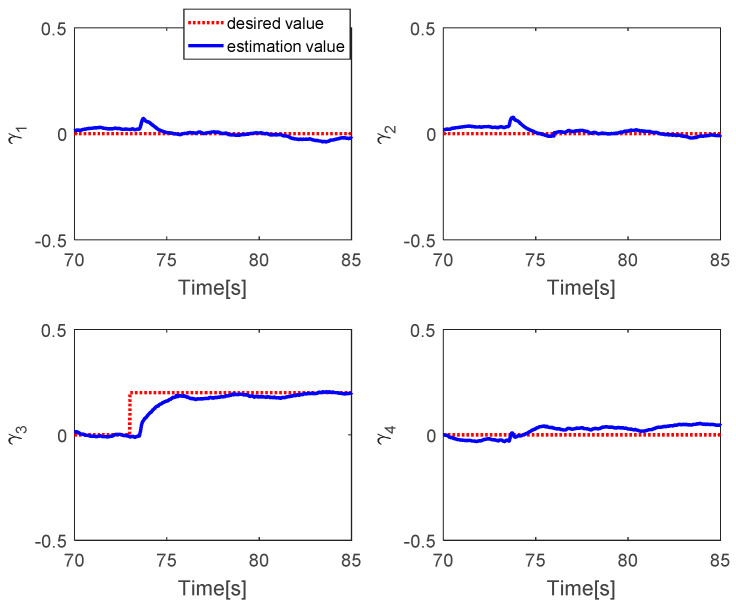
Fault estimation of each actuator.

The fault estimations of the control inputs are presented in Figure 9. This shows that the fault estimation values converge to the desired values. Figure 10 shows the fault estimation of each actuator. The fault estimation values of the first, second, and fourth actuators converge smoothly to zero, while that of the third actuator converges to 0.2.

#### 4.2.3. LoCE in the Three Actuators Using Magnitude Order Unbalance

**Figure 11:**
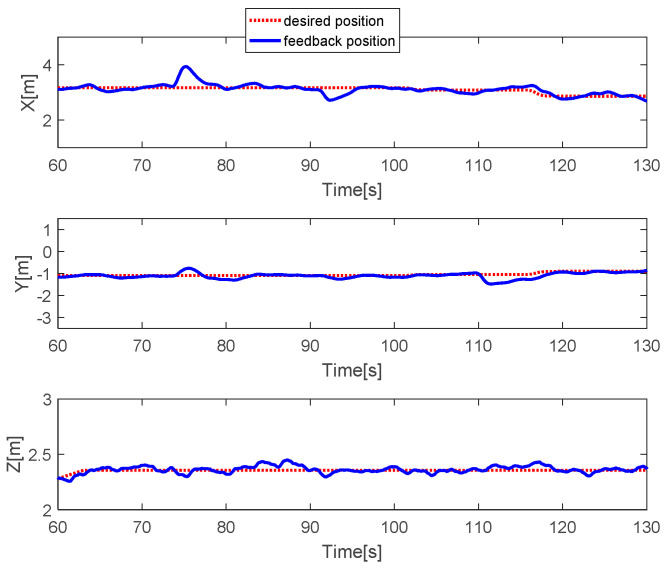
Horizontal and vertical movement.

The quadcopter system hovers at an altitude of 2.4 m. Faults with different magnitudes are injected into the third actuator with 22% LoCE at *t* = 73 s, a fourth actuator with 16% LoCE at *t* = 91 s, and a second actuator with 13% LoCE at *t* = 110 s. In Figure 11, the faults affect the horizontal movement. From *t* = 73 s to *t* = 80 s, when the third actuator is faulty, the x-direction deviates from the desired position by 60 cm, while the y-direction deviates from the desired point by 25 cm. From *t* = 90 to 110 s, when the fourth actuator is faulty, the x-direction deviates from the desired position with 50 cm, while the y-direction experienced a small deviation from the desired point by 10 cm. From *t* = 110 to 1300 s, when the second actuator is faulty, the y-direction deviates from the desired position by 60 cm, whereas the x-direction deviates slightly from the desired point. Compared to the horizontal movement, the vertical movement has a very small deviation when faults occur in each actuator.

**Figure 12:**
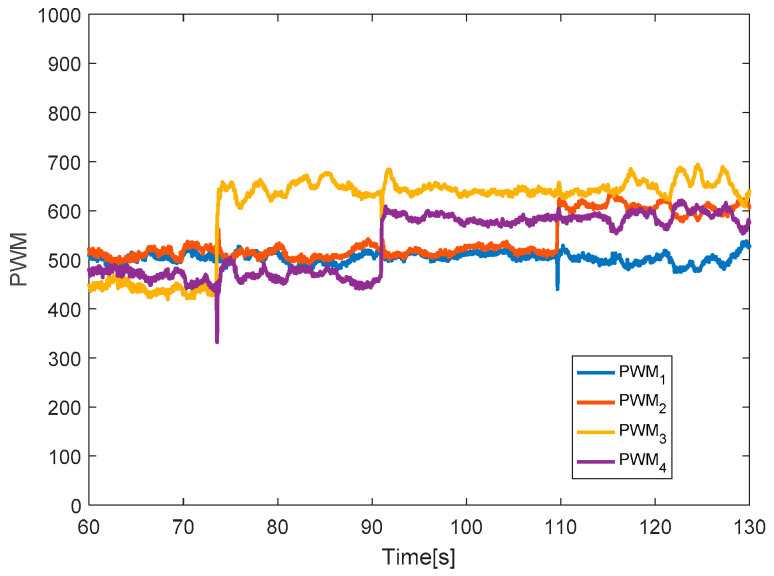
PWM inputs.

**Figure 13:**
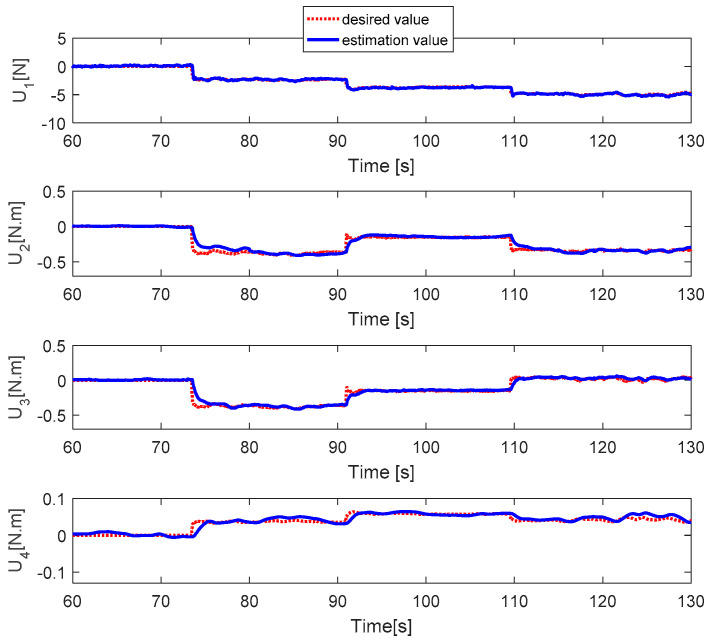
Fault estimation of control inputs.

**Figure 14:**
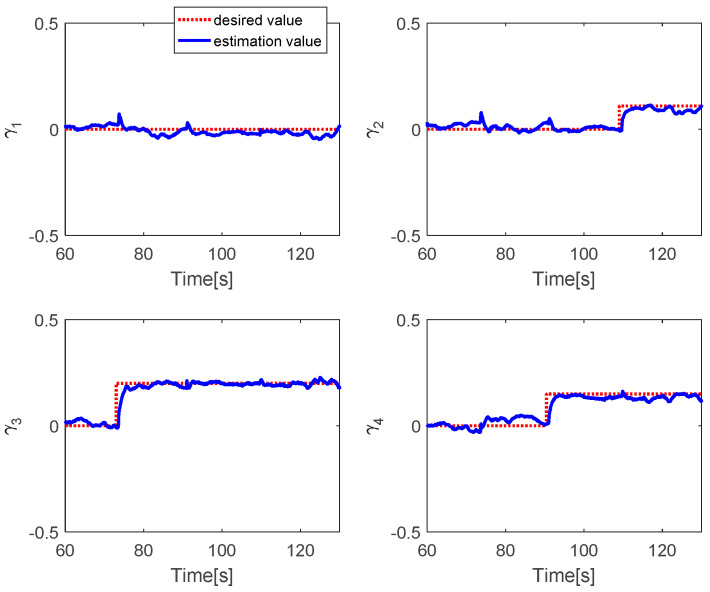
Fault estimation of each actuator.

Figure 12 shows the PWM inputs of all actuators. It is shown that before the fault is injected, all PWM inputs are almost the same. After faults occur in each actuator, the PID controller can recover the system by increasing the PWM input of the faulty actuator. Figure 13 reveals the fault estimations of the control inputs. This shows that the fault estimation values converge quickly to the desired values. Figure 14 describes the fault estimation of each actuator. The fault estimation values of the first, second, and fourth actuators converge to values of 0.22, 0.16, and 0.13, respectively, whereas the first actuator converges to zero.

#### 4.2.4. Loss of Control Effectiveness in Four Motors Using Magnitude Order Unbalance

**Figure 15:**
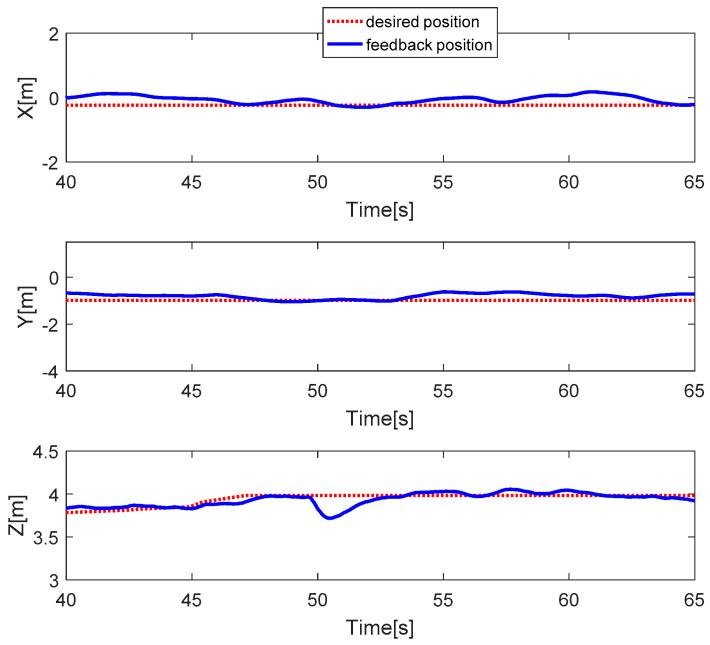
Horizontal and vertical movement.

**Figure 16:**
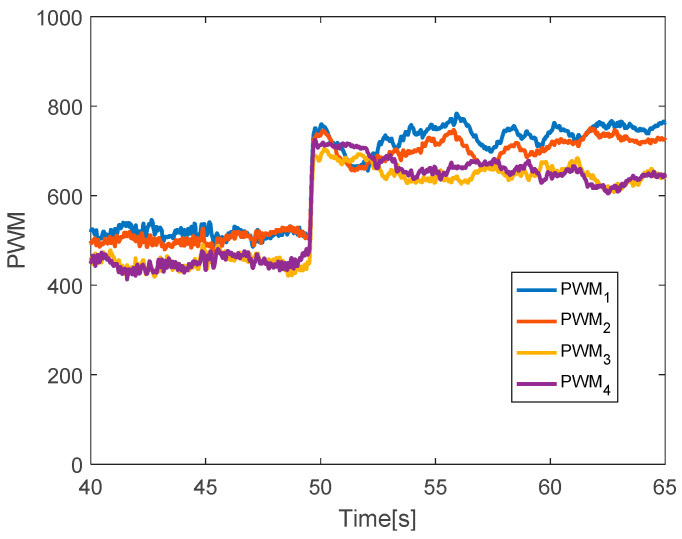
PWM inputs.

The quadcopter system hovers at the height of 3.8 m. The faults are injected into all actuators at time *t* = 50 s by 30% LoCE. As can be seen in Figure 15, the faults do not affect the horizontal movement, while the vertical movement drops to 0.4 m. The PWM inputs of all motors are shown in Figure 16. Before the faults are injected from time *t* = 0 to 50 s, all PWM inputs are almost the same. After faults occur in all motors, the PID controller can recover the system by increasing the PWM input of all motors.

**Figure 17:**
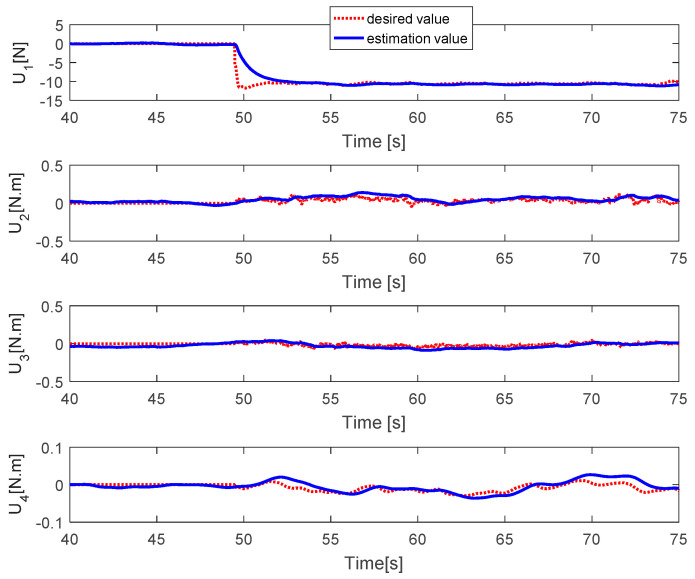
Fault estimation of control inputs.

**Figure 18:**
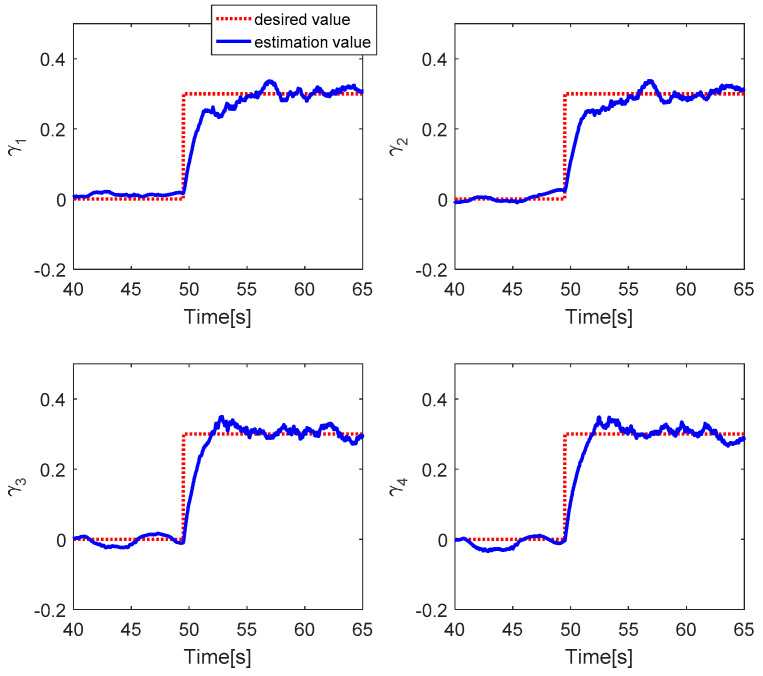
Fault estimation of each actuator.

Figure 17 presents the fault estimations of the control inputs. It shows that the fault estimation values converge quickly to the desired value. Figure 18 describes the fault estimation values of each actuator. It is shown that the fault estimation values of all motors converge to 0.3.

**Remark** **3.** *It should be mentioned that the previous observer-based fault diagnosis studies on quadcopter platform only consider the fault estimation of the roll, pitch, and yaw motion. In this article, the proposed method not only estimate the magnitude of the roll, pith, and yaw motion but also estimate the fault level of each actuator which is the differentiating point compared with other studies. The advantage of the presented fault estimation algorithm is the estimation of the actuator fault under model uncertainties.*

**Remark** **4.** *The presented method was applied and tested in the quadcopter platform. For other multicopters (with more than four actuators), the matrix* Π *is not invertible. A pseudo-inverse matrix can be applied but it may cause inaccuracy in the fault estimation algorithm, which is a limitation of this study.*

## 5. Conclusions

In this study, an intermediate observer is investigated for a fault estimation scheme of a quadrotor under actuator faults. The improved fault estimation algorithm and magnitude order unbalance method were validated through a flight test on the DJI F450 quadcopter platform. Four experiments are presented. The first two scenarios present the effectiveness of the magnitude order balance method under a faulty third actuator. The remaining scenarios are shown to evaluate the reliability of the presented algorithm in the presence of multiple faults. The results reveal that the investigated method can accurately estimate the fault magnitude of the roll, pitch, yaw, and thrust motion. Different from other studies on observer-based fault estimation, this work can obtain the loss of control effectiveness of each actuator (fault estimation of each actuator) in the presence of uncertainties. However, the limitation of this article is that it does not provide a reconfiguration controller to give a completely active fault tolerant control system. Moreover, the drawback of this method is that it may not applied for multicopters with higher than four actuators due to the pseudo-inverse matrix mentioned in Remark 3. Future work should discuss and implement the reconfiguration controller for the quadcopter system using the proposed fault estimation algorithm. Furthermore, the difficulty with the pseudo-inverse matrix problem is also discussed for a general multicopter platform. The investigated algorithm needs the Assumptions 3–4, but some mathematical models may not meet these assumptions. Further studies will consider the relaxing technique for these assumptions.

## Figures and Tables

**Table 1:** Fault estimation procedure.

**Input:** The control inputs and system matrices in (5) satisfying Assumptions 1–4.
**Step 1:** Choose small positive parameters of μ. **Step 2:** Solving the LMI matrix to obtain matrix L and α. **Step 3:** Fault estimation for roll, pitch, yaw, thrust motion in (14).
**Step 4:** Choose adjustment gain υ for magnitude order balance.
**Step 5:** Loss of control effectiveness of each actuator (Fault estimation of each actuator) in (38) is obtained.

**Table 2:** DJI F450 quadcopter parameters and observer design values.

Parameter	Description	Value
L	Arm length	0.225 m
$K_{th}$	Thrust coefficient	0.0087
$K_{d}$	Drag coefficient	$0.0055\times 10^{-2}$
m	Total mass	1.776 kg
$I_{x};\;\;I_{y};\;\;I_{z}$	Moments of inertia	$0.0035;\;\;0.0035;\;\;0.0055\;\;\;\mathrm{kg}{.m}^{2}$
$J_{r}$	Rotor inertia	$2.8\times 10^{-6}\;\;\mathrm{kg}{.m}^{2}$
υ	Adjustment gain	10
α	Constant	1
ε	Constant	0.02
η	Constant	1
